# Current Management of Oligometastatic Lung Cancer and Future Perspectives: Results of Thermal Ablation as a Local Ablative Therapy

**DOI:** 10.3390/cancers13205202

**Published:** 2021-10-16

**Authors:** Mario Ghosn, Stephen B. Solomon

**Affiliations:** Interventional Radiology Service, Memorial Sloan Kettering Cancer Center, 1275 York Avenue, New York, NY 10065, USA; ghosnm@mskcc.org

**Keywords:** lung cancer, cryoablation, metastases, microwave ablation, radiofrequency ablation

## Abstract

**Simple Summary:**

Up to 56% of lung cancer patients present with metastatic disease at initial diagnosis. Whereas these patients were historically deemed incurable, recent evidence supports the use of thermal ablation in stage IV non-small cell lung carcinoma with limited sites of metastasis (oligometastatic disease). In this review, we discuss the main results (local efficacy, overall survival, progression-free survival, safety and quality of life) of studies evaluating thermal ablation as a local ablative therapy for oligometastatic non-small cell lung carcinoma.

**Abstract:**

A growing body of evidence shows improved overall survival and progression-free survival after thermal ablation in non-small cell lung carcinoma (NSCLC) patients with a limited number of metastases, combined with chemotherapy or tyrosine kinase inhibitors or after local recurrence. Radiofrequency ablation and microwave ablation are the most evaluated modalities, and target tumor size <3 cm (and preferably <2 cm) is a key factor of technical success and efficacy. Although thermal ablation offers some advantages over surgery and radiotherapy in terms of repeatability, safety, and quality of life, optimal management of these patients requires a multidisciplinary approach, and further randomized controlled trials are required to help refine patient selection criteria. In this article, we present a comprehensive review of available thermal ablation modalities and recent results supporting their use in oligometastatic and oligoprogressive NSCLC disease along with their potential future implications in the emerging field of immunotherapy.

## 1. Introduction

### 1.1. Local Ablation Therapy for Oligometastatic Disease

Lung cancer remains the leading cause of cancer death in the United States; up to 56% of patients present with metastatic disease at diagnosis [1]. Non-small cell lung carcinoma (NSCLC) accounts for 84% of all primary lung cancer cases, with a poor prognosis for stage IV patients and a 5 year overall survival (OS) ranging from 0 to 10% [2].

NSCLC metastatic patients were historically deemed incurable, and the use of local ablative therapies (LAT)—namely, surgery, thermal ablation (TA) and stereotactic body radiation therapy (SBRT)—was restricted to symptom control. There is a growing body of evidence suggesting a role for LAT in improving OS and progression-free survival (PFS) in a subset of stage IV patients characterized by limited sites of metastasis (oligometastatic disease) [3,4,5] or of disease progression while under systemic therapy (oligoprogressive disease) [6,7].

### 1.2. Potential Advantages of Thermal Ablation over Other LAT

Whereas surgery was the most commonly used therapy for oligometastatic NSCLC [8], TA [9] and SBRT [10,11] have emerged as less invasive, more efficient, and safe alternatives for well-selected patients. Indeed, only one-third of patients are considered candidates for lung resection due to associated comorbidities [12].

TA of tumors consists of focal delivery of extremely high (typically >55 °C) or extremely low temperatures that induce irreversible cellular damage and, consequently, coagulation necrosis [13]. The therapy is widely accepted for lung metastases [14,15,16], renal cancer [17], and in both primary [18] and secondary liver malignancies [19]. Encouraging results were similarly reported for stage I [20,21,22] and oligometastatic NSCLC patients [23], with TA yielding survival outcomes comparable to surgery and SBRT in well-selected patients.

TA offers several advantages over other LAT modalities. It is a minimally invasive therapy that preserves lung parenchyma [24,25] and thus can be used in patients with an insufficient pulmonary function that are excluded from surgery and radiotherapy. A prospective single-arm international trial evaluating 183 lung tumors treated with radiofrequency ablation (RFA) in 106 patients showed no worsening in forced expiratory volume or forced vital capacity with regular visits over 1 year at follow-up [9]. Contrary to SBRT, TA is performed in a single session and can be repeated in case of recurrence [14], which is particularly relevant in the context of oligometastatic NSCLC patients, half of whom inevitably experience disease progression within 12 months [26]. TA has also been shown to improve patients’ quality of life by allowing a pause in chemotherapy (“chemoholiday”) for patients with primary colorectal cancer [27,28].

### 1.3. Indications of Thermal Ablation

Based on this evidence, TA was incorporated by the National Comprehensive Cancer Network into their guidelines for medically inoperable NSCLC patients with multiple lung cancers, no disease outside the chest, N0-1 status, limited (3 to 5) metastases, limited locoregional recurrence, limited (3 to 5) recurrence of distant metastases, and limited (3 to 5) sites of progression after therapy targeted to the epidermal growth factor receptor (EGFR) and anaplastic lymphoma kinase (ALK) [29]. In addition, the Society of Thoracic Surgery Expert Consensus on pulmonary metastasectomies recommended it as the first option for ipsilateral recurrence after surgery [30]. Authors also favored TA over stereotactic ablative radiotherapy (SABR) for 2–3 cm oligometastatic lesions in these high-risk patients, notably due to its better safety profile for pulmonary function [30]. However, TA has its own complications and limitations, in particular regarding tumor size and location [31], underlining the need for careful patient selection.

In this article, we review available thermal ablation modalities and current evidence supporting their use in oligometastatic and oligoprogressive NSCLC disease along with their potential future implications in the emerging field of immunotherapy.

## 2. Thermal Ablation Modalities

Commonly used technologies for TA include RFA, microwave ablation (MWA), and cryoablation, each with its own physical principles, but all delivered through one or more applicators placed percutaneously under imaging guidance [32].

### 2.1. RFA

During RFA, a high-frequency alternating current is applied through a needle electrode placed in the target tumor, and the electrical circuit is closed by a grounding pad [33]. The electrical current induces rapid vibration of dipole molecules, which results in a local temperature increase and, consequently, coagulation necrosis and complete destruction of the tumor [34].

One of the major drawbacks of RFA is the heterogeneity of heat deposition due to its dependence on a given tissue’s electrical [35] and thermal conductivity. Aerated lungs have a high impedance (low electrical conductivity), limiting the current flow generated by the RFA antenna, and a low thermal conduction, restricting heat diffusion to the surrounding tissues [36]. RFA is also susceptible to the “heat sink” effect, whereby thermal energy dissipates through a vessel or airway proximal to the electrode [37], which can result in insufficient ablation margins and local tumor recurrence. Ablation zone size is also limited to 4–5 cm maximum smallest diameter, as only one single probe can be activated at a time [38].

### 2.2. MWA

**Figure 1:**
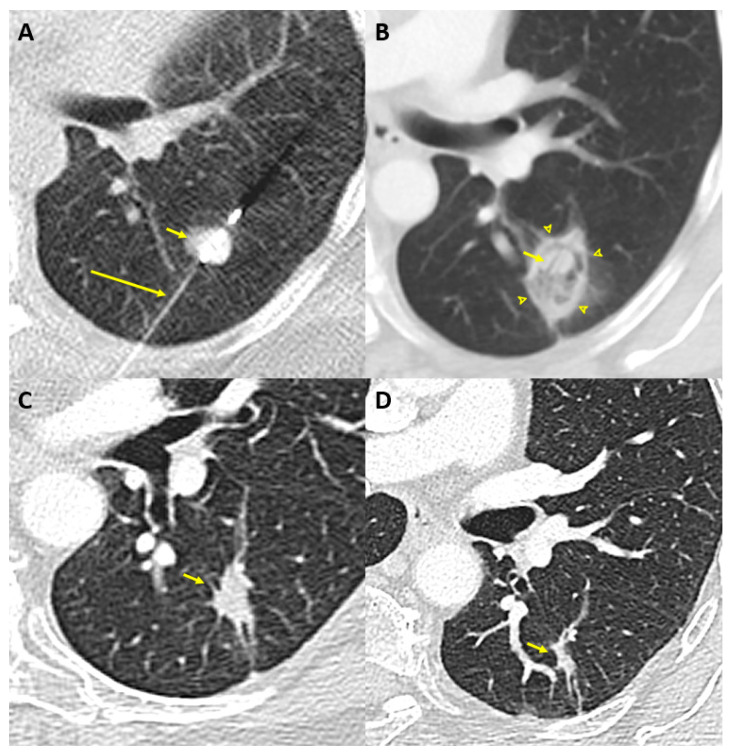
Example of MWA in a sixty-year-old woman with a 13 mm nodule in the apical segment of the left lower lobe. (**A**) Axial non-contrast chest computed tomography scan obtained during the MWA procedure shows the MWA probe (long arrow) placed percutaneously in the nodule (short arrow). (**B**) The computed tomography scan performed 6 weeks post-ablation shows a ground glass opacity (arrowheads) surrounding the nodule (short arrow) and corresponding to the ablation zone. In this case, technical success was confirmed with an ablation margin above 10mm. (**C**) Chest-computed tomography performed 13 months later confirmed the expected decrease in size post-ablation (short arrow). (**D**) Sixty-one months later, the post-ablation zone markedly decreased in size (short arrow), with no signs of local recurrence.

MWA uses oscillating electromagnetic waves (300 MHz to 300 GHz) emitted by the antenna to induce rapid flip motion of surrounding water molecules and increase local temperature [39]. Since it is less dependent on tissues properties, MWA reaches higher temperatures than RFA, and does so faster as well [40]. It also allows larger ablation zones by enabling simultaneous activation of multiple probes [41]. Given these advantages and its lower susceptibility to heat sink effect, MWA is increasingly being used for the treatment of lung and liver malignancies [42]. The main limitations of this technology are a lack of sphericity and only moderate reproducibility of the ablation zones [43]. An example of MWA in a sixty-year-old woman with a 13 mm nodule in the apical segment of the left lower lobe is shown in Figure 1.

### 2.3. Cryoablation

In contrast to RFA and MWA, cryoablation is based on the Joule–Thompson principle and uses cold to induce cell death. Multiple cycles of freezing and thawing are applied, and several probes can be used to treat larger tumors [44]. The obtained “ice ball” is visualized under computed tomography, allowing for real-time control of the ablation zone [45]. Cryoablation also offers the advantage of being less painful [46] and less harmful to collagen structures such as bronchi and can be performed under local anesthesia [47].

## 3. Definition of Oligometastases and Rationale for Thermal Ablation

The concept of oligometastases was introduced in 1995 by Hellman and Weichselbaum [48] to characterize an intermediate state between a localized stage and a widely metastatic disease. This hypothesis of a continuum process of cancer spread was corroborated by long-term survival observed after aggressive local treatment in patients with a limited number of metastases, such as those with colorectal liver metastases or sarcoma lung metastases and, more recently, oligometastatic NSCLC patients [49].

The definition of oligometastatic NSCLC is beyond the scope of this review and will be further developed in another article of this special edition. Briefly, oligometastatic patients can present in three main situations:

- Synchronous disease: patients that present at initial diagnosis with a limited number of metastases (mostly up to five) that are technically amenable to a radical treatment [50];
- Oligoresidual disease: patients who initially had multiple metastases and responded to systemic therapy with only limited metastases remaining, all amenable to a radical treatment [51];
- Metachronous disease (oligorecurrence): patients presenting with limited metastases after curative treatment to a locoregional disease, with an active disease now limited to the metastatic sites [52].

### 3.1. Adjuvant and Consolidation Therapy

Platinum-based doublet chemotherapy is the standard first-line therapy for most patients with advanced stage NSCLC, but is associated with only limited survival [53]. Because disease progression often occurs in original metastatic sites, it was hypothesized that the addition of LAT could help increase PFS and OS by eradicating the disease before it spreads, especially in patients presenting a limited number of metastases that did not progress after systemic treatment [54].

Improved survival in advanced stage NSCLC patients treated with TA in addition to chemotherapy or tyrosine kinase inhibitor (TKI) was reported in relatively small cohorts [55,56,57,58,59] and was recently confirmed in two larger analyses of the National Cancer Database. The first compared outcomes of stage IV NSCLC patients treated with surgery + systemic therapy, external beam radiation therapy (EBRT)/TA + systemic therapy, and systemic therapy alone [60]. After multivariable adjustment, surgery had the highest OS among three treatment groups, and patients in the EBRT/TA group (*n* = 9539) had higher OS than those in the systemic therapy only (*n* = 24513) group (*p* = 0.002). The second compared OS of stage IIIB and IV patients that received TA to a matched cohort that was not treated with ablation and found an improved OS [61].

### 3.2. Salvage Therapy after Local Recurrence

Given its safety profile and its repeatability, TA has been used for local recurrence following surgery, SBRT, or TA for NSCLC [62,63,64]. Additionally, it has proven to be a safe therapy for recurrences occurring in the radiation field [65].

A retrospective study evaluated outcomes of different LAT modalities, including SABR (*n* = 15), surgery (*n* = 10), and RFA (*n* = 6), as a salvage therapy for recurrence after SABR in early-stage NSCLC patients [66]. The 5 year OS rate was not statistically different between patients that had salvage treatment (57%) after isolated local recurrence (same lobe as the primary treated tumor) and patients that did not experience recurrence (54.9%, *p* = 0.65). OS after salvage therapy of isolated regional recurrence (hilum or mediastina) was similar to that of stage III disease. Despite selection bias (given that RFA patients were excluded from surgery and SABR), no patients experienced any grade ≥3 toxicity with RFA, compared to 6.6% in the SABR group and 40% for surgery. Moreover, local recurrence did not seem to differ among the three modalities. Interestingly, 99% of recurrences had been confirmed by biopsy, highlighting again a major advantage of image-guided TA, where the operator has the possibility to re-biopsy the tumor before ablation and thus provide valuable information for mechanisms of TKI resistance and disease management [7].

## 4. Materials and Methods

The aim of this mini review is to describe local efficacy, survival, and safety of thermal ablation in oligometastatic NSCLC patients, mainly in lung and liver metastases. A search of the English literature in Pubmed was performed for the period from 2000 to May 2021 using the following items: non-small AND (thoracic OR lung OR pulmonary OR liver) AND (ablation OR radiofrequency OR microwave OR cryoablation) AND (oligometastases OR oligometastasis OR oligoprogression OR metastases OR metastasis). The search was limited to human subjects and yielded 149 publications, and the papers most relevant to oligometastatic and oligoprogressive NSCLC patients treated with TA were included. Publications from selected article reference lists were also screened.

## 5. Results

The main outcomes of selected studies evaluating the role of TA in oligometastatic NSCLC patients are presented in Table 1 (prospective studies) [9,67,68,69] and Table 2 (retrospective studies) [55,70,71,72,73,74,75,76]. Only outcomes relevant to oligometastatic NSCLC patients were reported when available.

### 5.1. Survival

#### 5.1.1. Lung

Due to the heterogeneity of inclusion criteria (synchronous vs. metachronous disease; number, location, and size of metastatic sites; type of concomitant or adjuvant therapies; ablation techniques), reported median OS and PFS of oligometastatic patients treated with TA ranged between 14 to 41.6 months and 16 weeks to 23.5 months, respectively (Table 1 and Table 2). Some authors also reported survival results among other LAT modalities [7,67,68,75] or with stage I disease [9], making it difficult to extrapolate these results to the specific population of oligometastatic NSCLC patients treated with TA. However, it is noteworthy that these results are in line with those published by a systematic review on SBRT in oligometastatic extra-cranial NSCLC patients that reported a median OS ranging from 13.5 to 55 months and a PFS range of 4.4 to 14.7 months [77].

The association of TA with chemotherapy and/or TKI has been evaluated in different temporal combinations. For example, Ni et al. evaluated MWA as consolidation therapy for synchronous extracranial oligometastatic disease that did not progress after first-line therapy by EGFR-TKI. The addition of MWA was associated with a statistically significant increase in median PFS (16.7 vs. 12.9 months, *p* = 0.02) and median OS (34.8 vs. 22.7 months, *p* = 0.04) compared to TKI alone group [71]. This improvement in patient survival was confirmed in a single-phase II trial that also evaluated outcomes of LAT (including two patients treated with TA) as consolidation therapy following TKI or chemotherapy, with a median PFS of 23.5 months. Median OS was not reached after a median follow-up of 32.5 months. [67]. The authors found no differences in OS regarding age, sex, number of metastases, or presence of an actionable mutation [67].

In a different manner, Wei et al. conducted the first phase III randomized controlled trial that compared MWA followed by platinum-based doublet chemotherapy vs. chemotherapy alone in stage IIIB and IV NSCLC patients. Although the number of metastases was not reported, the authors showed an increase in PFS and OS in the MWA + chemotherapy arm. The difference remained statistically significant in the subgroup of stage IV patients, with a median PFS of 20.6 months and a median OS of 24.2 months (vs. 4.9 months and 12.4 months in the chemotherapy arm, respectively) [69]. A single arm phase II trial [68] showed that LAT to all known sites of disease can also be beneficial if performed before treatment by immunotherapy (pembrolizumab). Although only one patient had RFA, authors reported a median PFS of 19.1 months, compared to a historical median of 6.6 months (*p* = 0.005).

TA has also been used for local recurrence following surgery, SBRT or TA for NSCLC [62,63,64]. In selected studies, the 5 year survival rate was 55.7% post-surgical recurrence [72], and median OS reached 35 months for local recurrence after EBRT [74]. Tumor size ≤3 cm was again found to be associated with OS and PFS [72,73].

#### 5.1.2. Liver

Improved outcomes were also reported for metachronous or synchronous NSCLC liver metastases [76], with patients treated with TA + chemotherapy or TKI yielding higher PFS than those treated with only chemotherapy (11.0 vs. 5.2 months; *p* = 0.001). Although a similar trend was observed for OS, the difference was not statistically significant (27.7 vs. 17.7 months; *p* = 0.152). N3 nodal stage, ECOG status of one, and a number of three liver metastases were associated with a lower PFS. Another study by Tseng et al. [78] also associated ≤ five adenocarcinoma liver metastases with survival.

### 5.2. Oligoprogressive Disease

While revolutionizing the treatment of advanced stage NSCLC patients harboring EGFR and ALK mutations, TKI has nevertheless been challenged by the emergence of tumoral resistance, which shortens the duration of response and eventually leads to disease progression. Weickhardt et al. hypothesized that LAT could eradicate developing resistant clones before they spread and showed that surgery or radiotherapy could delay disease progression by 6 months in patients with EGFR or ALK mutations receiving erlotinib or crizotinib, respectively [79]. Interestingly, 49% of patients treated by erlotinib or crizotinib were eligible for this treatment strategy.

Selected studies reporting outcomes of TA in oligoprogressive NSCLC patients are summarized in Table 3 [7,75,80]. The three selected studies were retrospective, did not report follow-up time, and variously defined PFS1 and PFS2. In the largest retrospective study evaluating TA in this indication, Ni et al. [80] included 71 patients harboring EGFR mutation with extra-central nervous system oligoprogressive disease (defined as three or fewer metastases in one or two organs) after first-line therapy with erlotinib, gefitinib, icotinib, or afatinib. RFA or MWA were applied to all progressing sites of disease in association with the continuation of TKI and resulted in an extension of PFS by a median of 10.0 months, with a median OS of 26.4 months (range: 6–86 months).

### 5.3. Prognostic Factors

Appropriate patient selection is key for improved survival outcomes. Significant prognosis factors of OS include tumor size ≤3 cm [72,73], the addition of TA [70], and ECOG status [64,70]. Tumor histology may also influence survival outcomes, as suggested by Jiang et al. [75], who found a significant association between adenocarcinoma and PFS upon multivariate analysis. In one of the largest prospective series of RFA in lungs, which evaluated 1037 metastases from different primary histologies, mostly from colon, rectum, and kidney, the number of metastases was associated with OS [14]. However, given the lack of consensus, selected studies in oligometastatic NSCLC (Table 1 and Table 2) used different cut-offs, ranging between three and five metastatic sites. It is still too early to draw conclusions about the optimal number of metastases to treat. SABR-COMET-3 [81] and SABR-COMET-10 [82]—two randomized controlled phase III trials that will evaluate long-term survival outcomes for patients with 1 to 3 and 4 to 10 oligometastatic lesions, respectively—will provide insights to help define patient selection criteria. Finally, disease-free intervals have been associated with OS [14,23] and are a well-known factor of tumor biology that also must be considered.

### 5.4. Local Efficacy and Predictors of Recurrence

Local efficacy (absence of tumoral recurrence at the ablation site) ranged between 82% to 88% in prospective studies [9,69], and 55% to 92% in retrospective studies [55,74]. After repeat ablation, 5 year secondary local tumor progression even reached 5.4% [72]. Indeed, contrary to SBRT, TA can be repeated without additional morbidity, with similar survival outcomes compared to patients that did not experience tumor recurrence [64].

Tumor size <2–3 cm is a well-known factor of improved local control [22,83,84]. Different tumor sizes included in studies evaluating TA in oligometastatic NSCLC (Table 1 and Table 2) may thus explain variability in local control rates. For example, Cheng et al. included tumors up to 61mm in diameter, which possibly resulted in the high local progression rate of 45% at 1 year [74]. However, time to local progression for tumors <3 cm was 23 months (vs. 14 months for tumors >3 cm), similar to results by Schoellnast et al. that reported a time to local progression of 24 months for tumors <3 cm (vs. 8 months in larger tumors, *p* = 0.07) [73].

Local tumor control has also been shown to be associated with the absence of contact with a large vessel or a large bronchus [85,86] and complete coverage of tumor by the ablation zone with a margin of 5–10 mm [87,88]. In a matched case–control study [31], 48 patients with first local tumor progression after RFA were matched to a control group of 112 patients to control for nodule size (±5 mm tolerance), nodule number (≤2 vs. ≥3), and primary histological type (categories: colon, rectum, other). In the multiple regression model, only an ablation margin ≤5 mm remained a risk factor of local tumor progression. A distance ≤5 mm to a bronchus or a vessel >3 mm diameter was associated with insufficient ablation margins, and the authors point out the need to consider these factors in algorithmic decision-making.

Concerning tumor histology, Lencioni et al. reported no statistically significant difference in response between NSCLC and lung metastases [9], in line with a systematic review of prognostic factors of local recurrence after MWA in the lung [89] that found no association between local recurrence and the number of metastases, primary tumor histology, or disease-free interval. Interestingly, local recurrence was lower in recent studies and below 20% in patients enrolled after 2016, probably due to operators’ learning curves, technological advances in microwave devices, and improved methods for targeting and assessment [89].

Ablation modalities were also different: some authors used RFA [9], others preferred MWA [71], cryoablation [55], or a combination [76]. It is unclear whether this affected the ablation outcomes. Indeed, a retrospective study showed similar local efficacy for MWA and RFA, with a local recurrence rate of 3.7% and 7.6% (*p* = 0.32), respectively, after a mean follow-up of 488 +/− 407 days [90]. A recent meta-analysis of 53 studies conducted from 2010 to 2017 [91] reported complete ablation rates of 86.1% and 81.1% and median local tumor PFSs of 22.0 months and 31.5 months for RFA and MWA, respectively (*p* = 0.249). No statistically significant difference was observed for 1, 2, 3, 4 and 5 year local tumor PFS. Subgroup analysis showed no difference in median OS between RFA (28.4 months) and MWA (24.4 months) groups. In liver, no difference was observed in terms of local efficacy between RFA and MWA, although long-term recurrence seemed lower with MWA [92].

### 5.5. Safety and Quality of Life

The preservation of patients’ quality of life is a major strength of TA [9]. Most reported complications were minor, self-limited [9,69,70,71,72], and did not prolong hospital stay, with a reported median of 1 to 3 days [9,73]. Only 10% to 29% of pneumothoraxes required a chest tube placement [69,73], often removed in 1 day without altering the patients’ quality of life, as demonstrated in the ECLIPSE trial [93], which questions its clinical significance [94]. In comparison, a chest tube is often placed following lung surgery, entailing longer durations and longer hospital stays [95]. Common complications include pain, pleural effusion not needing treatment, and self-limited intrapulmonary hemorrhage [9,69,70,71,72]. In addition, TA did not alter the course of systemic therapies, with similar adverse events found when MWA was combined with chemotherapy (but not in the chemotherapy alone group [69]), and no reported TKI discontinuation because of MWA [71].

In summary, TA’s local safety and efficacy has been well-proven in oligometastatic NSCLC, given careful selection of patients. Important selection criteria include tumor size <3 cm, distance from large vessels and airways, and ablation margins >5 mm. Whereas MWA and cryoablation offer some interesting advantages over RFA, choice of ablation modality still depends on the operator’s experience, since none of these techniques have proven inherently superior [91,92,96].

### 5.6. Comparison to Other LAT Modalities

Comparisons of different LAT modalities in terms of survival outcomes are limited by the heterogeneity of published studies. Moreover, patients evaluated in TA series often had several comorbidities and were contra-indicated to surgery or SBRT, making any comparison subject to a major selection bias. Although randomized controlled trials are needed to delineate the role of each therapy in the management of oligometastatic NSCLC patients, a growing body of evidence suggests that TA is not inferior to lung resection or SBRT in carefully selected patients, as shown by Hasegawa et al. for oligometastatic colorectal lung metastases ≤3 cm [97], or by a large series on lung metastases [14] that yielded similar local control rates and similar OS.

These results are further supported by several studies that retrospectively compared TA to lung resection [98,99,100] and SBRT [101] in stage I NSCLC selected patients. In a comparison of early-stage NSCLC patients treated with SBRT (*n* = 14,651) and TA (*n* = 1141), SBRT showed similar OS than TA for tumors ≤2 cm [102], highlighting again the need for careful patient selection.

In sum, although it is acknowledged that most of the literature evaluating TA in oligometastatic NSCLC consists of small series, mostly retrospective without a controlled arm, there is enough evidence to suggest a clinical benefit to treating oligometastatic NSCLC patients with TA, with an efficacy similar to surgery and SBRT. Combining these therapies might be interesting in some cases, and choosing the optimal LAT for each individual patient requires a multidisciplinary team that includes thoracic surgeons, oncologists, and interventional radiologists experienced in lung ablations; indeed, a multidisciplinary approach has been repeatedly proposed [29,30], as collaboration has been shown to increase adherence to clinical guidelines and may also improve patients’ quality of life [103].

**Figure 2:**
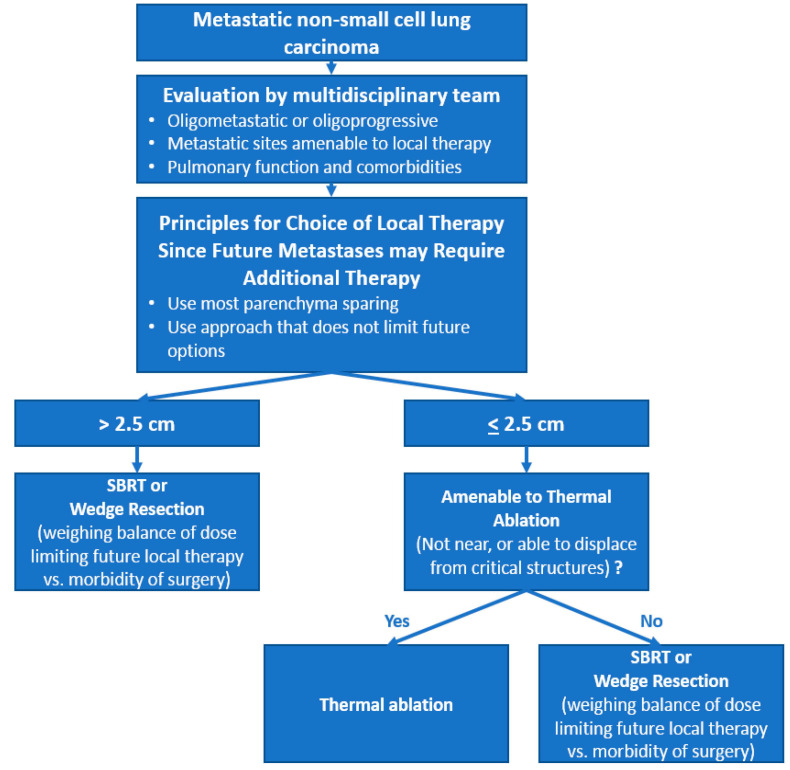
Proposed decision algorithm for local ablation therapy of oligometastatic non-small cell lung carcinoma.

Based on the results of this review, previously published recommendations on lung metastases by Handy et al. [30] and Najafi et al. [104], and the recent Society of Thoracic Surgeons webinar on pulmonary metastasectomy [105], we propose an algorithm to help choose the optimal local ablation therapy among surgery, SBRT and TA, illustrated in Figure 2.

## 6. Future Perspectives in the Era of Immunotherapies

The antibodies anti-programmed death ligand 1 (PD-L1) and anti-programmed death 1 (PD-1) have recently emerged as new therapeutic options for non-oncogene-driven advanced stage NSCLC patients [106]. Despite impressive clinical results, the majority of patients develop primary or secondary resistance, stressing the need for new strategies to overcome these shortcomings [107]. By boosting the immune response, TA may play a major role in this setting, in particular when associated with immune checkpoint inhibitors (ICI).

Contrary to surgical resection, TA not only eradicates the tumor locally but also leaves tumor neoantigens and associated antigens in situ [13]. Such an approach can activate a systemic immune response, which, in turn, can eliminate distant no-target metastases. Since this so-called “abscopal effect” is rarely observed and rarely reproduced, studies focus instead on associating TA and immunomodulation in order to enhance therapeutic efficacy and provide a sustainable anti-tumoral response.

Several pre-clinical studies supporting this hypothesis show improved survival and tumor control with combination therapies [108]. Among different TA modalities, cryoablation has received the most interest, as higher pro-inflammatory cytokine levels (including interleukin 1 and interleukin 6) observed after cryoablation suggest a greater immune response than that of RFA and MWA [13]. Additionally, cold injury induces less protein denaturation then heat-based TA and preserves intracytoplasmic content, thus potentially releasing more antigens into systemic circulation [13]. An ongoing phase II clinical trial (ClinicalTrials.gov, identifier: NCT03290677) is evaluating the safety and feasibility of cryoablation of growing tumors in stage IV lung cancer patients progressing under ICI; the authors will assess the radiological response rate as a secondary outcome. The CRYOVATE trial (ClinicalTrials.gov, identifier: NCT04793815) will evaluate the role of cryoablation in advanced/metastatic or unresectable NSCLC patients (PD-L1 ≥50%) that will be subsequently treated with pembrolizumab (anti-PD-1) monotherapy. Regarding safety, a recent study evaluating the combination of TA and ICI [109] reported no adverse events among all 12 treated NSCLC patients.

Research in this field is, however, in its beginnings, with very limited results in humans and many questions remaining to be answered, such as those regarding the types of induced immune response, appropriate ablation modality, and the most beneficial and synergistic combinations [110].

## 7. Conclusions

In conclusion, thermal ablation is a minimally invasive therapy that offers some advantages over surgery and radiotherapy. Although randomized studies are still lacking, a growing body of evidence supports its use in NSCLC patients with a limited number of metastases, combined with chemotherapy or tyrosine kinase inhibitors or after local recurrence. A target tumor size <3 cm (and preferably <2 cm) remains a key factor of technical success and clinical efficacy. Optimal patient management requires a multidisciplinary approach, and further randomized controlled trials are needed to help refine patient selection criteria and treatment modalities.

## Figures and Tables

**Table 1:** Selected prospective studies evaluating thermal ablation for oligometastatic NSCLC patients.

Ref (Year)	Study Design	No	TA	Sites Treated with TA	Indication of TA	Mean Tumor Size (Range)	Median FUP (mo)	Median PFS (mo)	Median OS (mo)
Lencioni (2008) [9]	Single-arm, phase II	106 (20 NSCLC with metastases or recurrence)	RFA	Lung	≤3 tumors per lung ≤3.5 cm Patients not candidate for surgery, radiotherapy or chemotherapy Recurrence after surgery or multiple lung metastases	22 mm * (7–30)	-	-	1 y and 2 y OS of 70% and 48% *
Arrieta (2019) [67]	Single-arm, phase II	37 (2 treated with TA)	RFA	-	≤5 metastases (including CNS) Synchronous SD or PR after 4 cycles of chemotherapy or TKI	-	32.5	23.5 **	NR **
Bauml (2019) [68]	Single-arm, phase II	45 (1 treated with TA)	-	-	≤4 metastases Previous LAT to all metastatic sites Synchronous and metachronous	-	25	19.1 **	41.6 **
Wei (2020) [69]	Phase III RCT	148 (MWA+ chemo group) vs. 145 (chemo only)	MWA	Lung	Stage IIIB or IV Number of metastases not defined TA performed on the primary tumor or the largest pulmonary metastases in case of previous surgery	36 mm (10–130)	13.1 vs. 12.4	10.3 vs. 4.9	NR vs. 12.4

* Results reported for all 33 NSCLC patients (13 patients with stage I and 20 patients with metastases or recurrence). ** Results reported with other LAT (radiotherapy and/or surgery). Abbreviations: Chemo = chemotherapy; CNS = central nervous system; FUP = follow-up; LAT = local ablative therapy; mo = months; MWA = microwave ablation; No = number of patients included in the study; NR = not reached; NSCLC = non-small cell lung carcinoma; OS = overall survival; PFS = progression-free survival; PR = partial response; RCT = randomized controlled trial; Ref = reference; RFA = radiofrequency ablation; SD = stable disease; TA = thermal ablation; TKI = tyrosine kinase inhibitor; 1 y = 1 year; 2 y = 2 year.

**Table 2:** Selected retrospective studies evaluating thermal ablation for oligometastatic NSCLC patients.

Ref (Year)	No	TA	Sites Treated with TA	Indication of TA	Mean Tumor Size (Range)	Median FUP	Median PFS	Median OS
Bang, (2012) [55]	31	Cryo	Lung, liver, superficial, paraaortic, adrenal, bone	<7 cm ≤5 metastases per organ site 84% treated with various chemotherapy and/or TKI regimens at some point before or after TA	31 mm (NA)	Mean = 11 mo	-	15.9 mo, 1-y OS of 53%
Li (2013) [70]	49	RFA	Lung	PR or SD after first line chemotherapy ≤5.0 cm ≤3 tumors >1.0 cm away from hilum and major bronchi or vessels	29 mm (14–50)	19 mo	16 weeks	14 mo
Ni (2020) [71]	86 (34 treated with MWA)	MWA	Lung, liver, bone, adrenal gland, chest wall	Synchronous extra-cranial disease No progression after EGFR-TKIs ≤5 metastases TA performed on primary tumors and oligometastatic lesions (consolidation) compared to patients receiving only TKI	29 mm (1–56)	36 mo	16.7 mo vs. 12.9 mo	34.8 mo vs. 22.7 mo
Kodama (2012) [72]	44	RFA	Lung	Post-surgical recurrence (initial stage I to IV) in ipsilateral (63.6%) or contralateral (36.4%) lung Contra-indication to surgery ≤5 metastases No extrapulmonary metastases *	17 mm (6–40)	Mean = 28.6 mo	-	1 y, 3 y, 5 y OS of 97.7%, 72.9%, 55.7%
Schoellnast (2012) [73]	33	RFA	Lung	Recurrence following surgery, chemotherapy, and/or radiotherapy Single lung lesion (except one patient with lung metastases)	28 mm (10–75)	24 mo	8 mo	21 mo
Cheng (2016) [74]	12	RFA, MWA	Lung	Local recurrence following radiotherapy (initial stage of disease: I to III) (in the radiation field) Contra-indication to radiation or surgery RFA was used for 2 patients and MWA for 10 patients	34 mm (17–61)	Mean = 19 mo	-	35 mo
Jiang (2019) [75]	64 OM (5 treated with TA)	RFA	Liver	≤5 liver metastases ≤5 cm LAT only on metastatic tumors LAT with EGFR-TKI compared to EKFR-TKI monotherapy	-	-	12.9 mo ** vs. 7.9 mo	36.8 mo ** vs. 21.3 mo
Zhao (2020) [76]	61 (21 treated with TA)	RFA, MWA	Liver	≤5 extracranial metastases ≤3 liver metastases, ≤5 cm After 4 cycles of chemotherapy or TKI TA before or concurrently with systemic therapy, compared to systemic therapy alone	24.4 mm (NA)	36.4 mo	11.0 mo vs. 5.2 mo	27.7 mo vs. 17.7 mo

* One patient had also liver and spleen metastases that were treated by RFA with curative intent. ** Results reported with other LAT (radiotherapy and surgery). Abbreviations: Cryo = Cryoablation; EGFR = epidermal growth factor receptor; FUP = follow-up; LAT = local ablative therapy; mo = months; MW A= microwave ablation; NA = not available; No = number of patients included in the study; NSCLC = non-small cell lung carcinoma; OM = oligometastatic; OS = overall survival; PFS = progression-free survival; PR = partial response; Ref = reference; RFA = radiofrequency ablation; SD= stable disease; TA = thermal ablation; TKI = tyrosine kinase inhibitor; 1 y = 1 year; 3 y = 3 year; 5 y = 5 year.

**Table 3:** Selected studies evaluating thermal ablation for oligoprogressive NSCLC patients.

Ref (Year)	No	TA	Sites Treated with TA	Indication of TA	Mean Tumor Size (Range)	Median PFS1 (mo)	Median PFS2 (mo)	Median OS (mo)	PFS Definitions
Yu (2013) [7]	18 (2 treated with TA)	RFA	Lung	<5 metastases (except one patient) Progression on EGFR-TKI RT and surgery also used to treat various sites of disease progression (lung, lymph node, adrenal gland)	-	10 *	22 *	41 *	PFS1 = from local therapy to progression PFS2 = from local therapy to change in systemic therapy
Jiang (2019) [75]	71 OP (8 treated with TA)	RFA	Liver	≤5 liver metastases ≤5 cm LAT only on metastatic tumors LAT with continuous EGFR-TKI compared to switching therapy	-	-	13.9 * vs. 9.2	28.3 * vs. 17.1	PFS1 = from TKI to first progression or death PFS2 = from TKI to off-TKI progression or switching therapy
Ni (2019) [80]	71	RFA, MWA	Lung, liver, adrenal, pleura, lymph node	≤3 metastases Extra-cranial progression ≤3 extra-CNS organs TA for all progressive lesions with continued EGFR-TKI treatment	33 mm (10–105)	11.8	10.0	26.4	PFS1 = from TKI to first progression PFS2 = from first progression to second progression after TA

* Results reported with other local ablation therapies (radiotherapy and/or surgery). All selected studies were retrospective and did not report follow-up time. Abbreviations: EGFR = epidermal growth factor receptor; FUP = follow-up; mo = months; LAT = local ablative therapy; MWA = microwave ablation; No = number of patients included in the study; NSCLC = non-small cell lung carcinoma; OP = oligoprogressive; OS = overall survival; PFS = progression-free survival; Ref = reference; RFA = radiofrequency ablation; TA = thermal ablation; TKI = tyrosine kinase inhibitor.
